# Socio-technical challenges in accessing antenatal services during pregnancy complications in Ecuador and the opportunities for digital health

**DOI:** 10.1177/20552076251343684

**Published:** 2025-06-09

**Authors:** Lorena Carlo, Eduardo Cruz, Francisca Antonella Carpio-Arias, Martin Baca, Maria Paula Jaramillo Carlo, Valeria Carpio-Arias, William Waters, Nicola Mackintosh, Nervo Verdezoto

**Affiliations:** 1 Department of Health Informatics, Rutgers University, Piscataway, USA; 2 Facultad de Ingeniería en Electricidad y Computación, 27883 Escuela Superior Politecnica del Litoral, Guayaquil, Ecuador; 3 Instituto Tecnológico José Ortega y Gasset, Riobamba, Ecuador; 4 Institute for Research in Health and Nutrition, 27902 Universidad San Francisco de Quito, Quito, Ecuador; 5 27887 Facultad de Ciencias de la Salud, Universidad Católica de Santiago de Guayaquil, Guayaquil, Ecuador; 6 School of Public Health, Escuela Superior Politécnica de Chimborazo, Riobamba, Ecuador; 7 Department of Population Health Sciences, 4488 University of Leicester, Leicester, UK; 8 School of Computer Science and Informatics, 2112 Cardiff University, Cathays, Cardiff, UK

**Keywords:** Antenatal care, digital health, high-risk pregnancies, socio-technical constraints, organizational constraints

## Abstract

**Objective:**

In Latin America, there is an increasing prevalence of pregnancy complications, leading to poor maternal and neonatal outcomes. Although essential antenatal health services are available to diagnose and treat pregnancy-related complications, their uptake is low and results in many Latin American women not receiving adequate antenatal care. This study aimed to understand the challenges experienced by women managing high-risk pregnancies while interacting with antenatal services and the perceived value of digital health in supporting their care practices.

**Methods:**

This qualitative case study collected data through eight focus group discussions with 43 pregnant women with complications, and 33 semi-structured interviews with healthcare professionals across different levels of the healthcare infrastructure in public and private hospitals and health subcenters in three Ecuadorian cities.

**Results:**

Our findings uncovered a number of material, spatial, technical, organizational, and everyday life constraints that negatively impacted women's access and experiences with antenatal services during pregnancy complications. Unintended consequences were also discovered in the fragmented Ecuadorian healthcare system, including extra data work, duplication of information, incomplete medical records and delayed diagnosis. Another important finding was the necessity for emotional support for healthcare professionals and pregnant women dealing with complications. Healthcare professionals and pregnant women perceived value in digital health that can support women's self-care practices as well as the communication, coordination and information management within and across healthcare institutions to improve antenatal care.

**Conclusions:**

This study provides a contextual understanding of the socio-technical challenges and constraints that affect the access and uptake of antenatal services during pregnancy complications in Ecuador. We discuss the potential of digital health to support both women and healthcare professionals’ efforts while caring for pregnancy complications and the need for taking a sociomaterial approach to scope digital health opportunities in antenatal care.

## Introduction

Maternal health is one of the biggest public health concerns around the world. In 2017, approximately 295,000 women died from causes related to pregnancy and childbirth, and 94% of these occurred in low- and middle-income countries (LMICs) according to the World Health Organization.^1^ Major direct causes of maternal mortality include several complications during pregnancy and birth or within 42 days after birth, including severe bleeding, pregnancy-induced hypertensive disorders (e.g. pre-eclampsia and eclampsia), abortion, sepsis, maternal infections, and obstructed labor.^1^ In addition, pre-existing conditions and non-communicable diseases during pregnancy make the situation more complex and indirectly influence maternal mortality. These include HIV, cancer, respiratory disorders, diabetes, and cardiovascular diseases.^2^

Previous research has investigated the existing challenges women face while accessing antenatal services in LMICs^3,4^ for example, in Rwanda,^5^ Ethiopia,^6^ Kenya,^7^ Lebanon,^8^ India,^9,10^ and Pakistan.^11^ Poorly equipped healthcare infrastructures,^2^ misalignment between antenatal care provision and the local context^3^ together with the increasing socio-cultural and economic inequalities,^12,13^ limit the utilization of antenatal care services (ANCs) in LMICs. Yet, little is known about the challenges women face while accessing and utilizing antenatal services in Latin America (LATAM),^14–16^ especially for women managing pregnancy complications.^17,18^ Although the Latin American and Caribbean regions have made considerable progress in improving maternal health in recent decades, maternal mortality rates have only improved from 96 female deaths per 100,000 live births in 2000 to 74 female deaths per 100,000 live births in 2017.^1^ Large inequalities in access and quality of care remain^19,20^ and pregnancy complications such as gestational diabetes and hypertensive disorders^21,22^ together with an increasing prevalence of anemia and obesity,^23–25^ are major contributors to poor maternal outcomes for women and their unborn babies in LATAM countries.

Although antenatal services offer opportunities to identify and treat pregnancy complications, several constraints negatively impact timely access and adequate provision of antenatal care. Previous studies have reported material constraints such as under-resourced facilities, poor availability of essential medications and equipment, shortage of water and power, and poor referral systems.^26^ Structural constraints include negative attitudes of healthcare workers,^27^ poor knowledge and skills of healthcare staff,^26^ long waiting times at health facilities,^26–28^ delays in booking appointments,^29^ or no available appointments.^27^ Accessibility to healthcare centers is another barrier. For example, previous studies indicate that pregnant women skip antenatal care due to lack of transportation or having to walk long distances to health centers^26,30^ or inability to pay for transportation.^30,31^ Other barriers to antenatal care include everyday life constraints such as house chores, childcare tasks^32,33^ and work schedule restrictions.^32,34^ Knowledge-related constraints further hinder pregnant women from receiving adequate antenatal care. Examples include low health literacy in pregnant women, not recognizing the importance of antenatal care,^26,32,35^ not knowing where to go to receive adequate care,^27^ language barriers,^32^ and low level of education.^33,34^

Although maternal health research has tended to focus on pregnancy and post-birth general information needs,^36^ more qualitative research is needed looking at pregnant women's specific needs/practices related to self-diagnosis and help seeking^37^ and barriers and facilitators for the use of digital health to support pregnancy-related complications,^38^ especially in LATAM contexts.^18,39^ Thus, it is necessary to further understand the challenges pregnant women and healthcare professionals face when accessing and providing antenatal services during pregnancy complications in LATAM contexts. This qualitative study aimed to further understand the barriers that hinder the utilization of antenatal care,^40^ a critical topic when considering the fragmented care structures in Ecuador, and the potential role of digital health to support antenatal care and pregnancy complications,^37,39,41,42^ in the Ecuadorian context.

### Antenatal care services in Ecuador

The provision of antenatal services is fragmented and segmented across public and private institutions.^43^ The public sector includes the Ministry of Public Health (MSP), the Ministry of Economic and Social Inclusion (MIES), the health care services provided by municipalities, and the social security institutions such as the Ecuadorian Social Security Institute (IESS), the Armed Forces Social Security Institute (ISSFA), and the National Police Social Security Institute (ISSPOL). Although the MSP offers healthcare services to the entire population, including antenatal care, MIES, and the municipalities provide health programs and medical services to the uninsured population. Social security institutions administer numerous programs to cover the affiliated working class, including maternal health services. The private sector includes for-profit organizations and non-governmental organizations that provide access to antenatal care through hospitals, clinics, doctor offices, pharmacies, prepaid medical insurance, general and specialized hospitals.^43–45^

**Figure 1.**
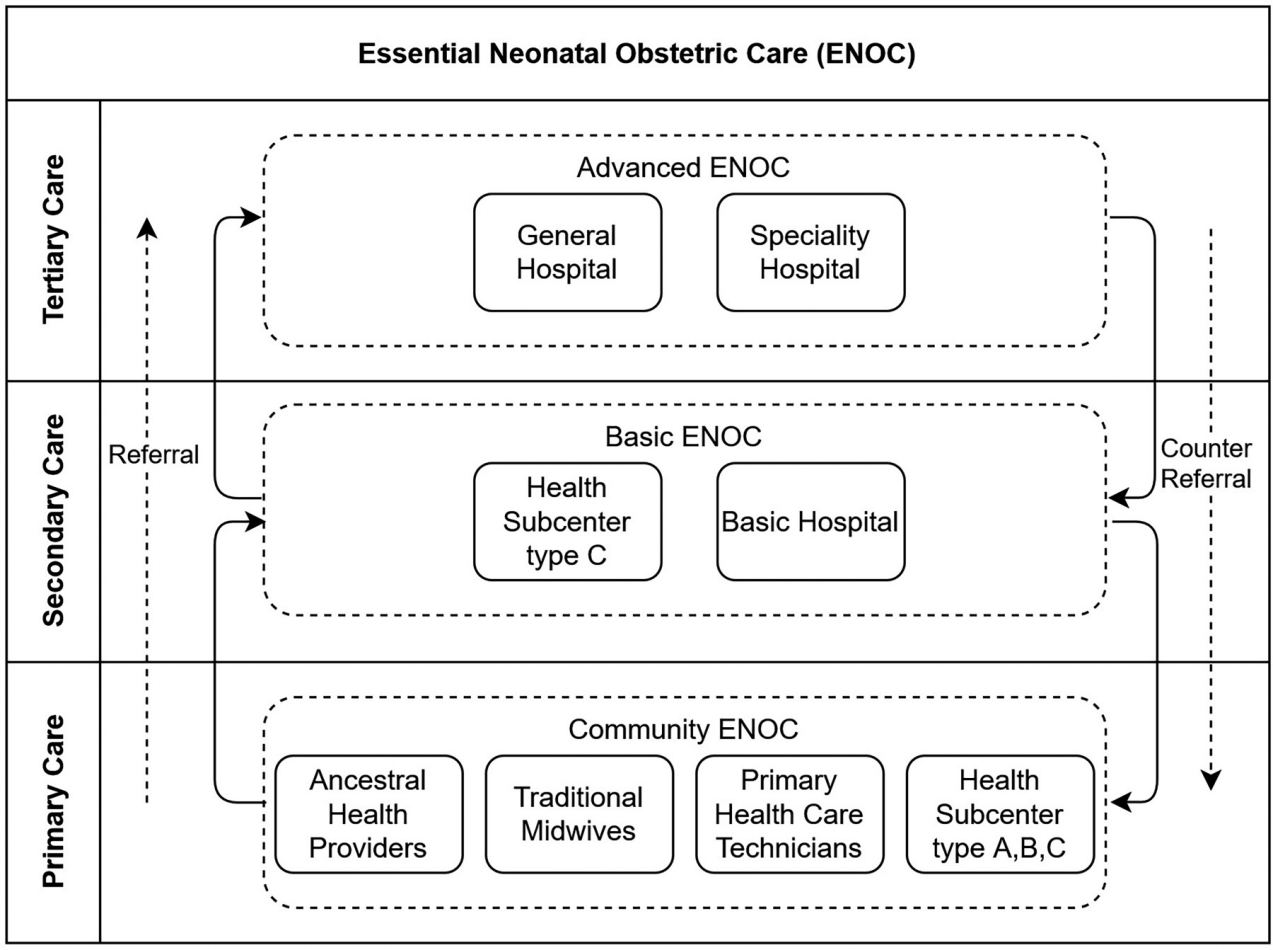
The essential neonatal obstetric care (ENOC) healthcare delivery system.

To improve the provision of obstetric care and neonatal health services, the public healthcare system coordinated by the MSP has developed an Essential Neonatal Obstetric Care (ENOC) policy, which establishes a set of strategies and actions to reduce the maternal and neonatal mortality ratio.^46^ Figure 1 shows an overview of the ENOC healthcare delivery system with three levels: community, basic, and advanced. The community level provides a local model for organizing and coordinating maternal and neonatal care services led by MSP primary health subcenters type A, B, and C, as well as the involvement of different community health providers such as traditional midwives (e.g. “parteras” or indigenous birth attendants)^47–49^ and ancestral health providers (e.g. community, traditional or indigenous healers).^50–52^ The competencies of the community level include identifying pregnant women at risk of pregnancy complications, promoting antenatal care programs, and monitoring compliance and prevention through home visits by family doctors. The health subcenters A, B, and C provide essential services to promote prevention, recovery, and palliative care through nursing, general medicine, odontology, psychology, and pharmacy. Health subcenters B and C also provide nutritional support, social work services, auxiliary diagnostic services in clinical laboratories, essential imaging, and audiometry optionally. Health subcenter type C provides gynecology, pediatrics, short-stay maternity units, and emergency visits. The main differences between these health subcenters lies in the range of specialties and the level of care available at each type, with type A focusing on primary healthcare, type B incorporating additional diagnostic and support services, and type C offering the most comprehensive and specialized medical care.^53^ The health subcenter providers can make referrals for timely attention to complex problems and emergencies.

Primary hospitals and the type C health subcenters are parts of the basic level. They offer essential obstetric care services focusing on medical cases of medium complexity and ensure compliance with at least five antenatal checkups, complementary tests, magnesium sulfate for pre-eclampsia, antibiotics (in the event of premature rupture of membranes), uterine inhibitors, and corticosteroids in threatened preterm labor up to 35 weeks. The basic level can make counter-referrals to the community level, and in the event of more significant complications, refer the patient to the advanced level. The advanced level includes the general and specialized hospitals that provide adequate healthcare services for major complications when emergency or scheduled appointments require clinical and surgical hospitalization, also providing gyneco-obstetric and neonatal care services (inpatient, outpatient, and day patient).

### Pregnancy complications in Ecuador

Pregnancy complications are a complex public health concern. They are the result of multiple factors shaped by social context. Ecuadorian women of low socioeconomic status, in both rural and urban areas and from diverse ethnic backgrounds, are among those who have lower access to health services,^54,55^ and are particularly at risk of pregnancy complications (e.g. gestational diabetes, hypertensive disorders^56,57^ and maternal infections^58^). The main risk factors include maternal age (under 15 years old, or older than 40 years old),^59^ women's lack of health knowledge, poor family support, lack of access, and inadequate antenatal care.^60,61^ Pregnancy complications have increased the risk of poor perinatal outcomes, and the number of cesarean sections and admissions to the intensive care unit.^58,61^ Although the maternal mortality ratio (MMR) of Ecuador has gradually decreased from 100 deaths per 100,000 live births in 2003 to 59 deaths per 100,000 live births in 2017,^1^ there are internal differences where the MMR can be higher.^59^ In particular, the Chimborazo province has the highest mortality rate, 98.12 per 100,000 live births, almost double the national average in 2018.^62^ Moreover, socio-cultural inequalities (e.g. poverty, low education) and the top-down medicalized model of care have influenced maternal mortality regardless of geographical location.^59^ In addition, Ecuadorian women are increasingly at risk of multiple forms of malnutrition (anemia, obesity, underweight, and overweight), making the situation more complex,^23^ especially when multiple health complications coexist. Although antenatal care providers play a crucial role in identifying and helping pregnant women manage their complications, pregnant women find it difficult to access and engage with different types of antenatal services.^34^ Factors associated with insufficient or lack of use of services include lower educational level, greater number of pregnancies, occupation in the agriculture or livestock sector, and being from minority ethnic groups.^34^ However, little is known about the challenges pregnant women face while accessing and interacting with antenatal services during complications.

### Digital health for maternal health in LATAM

According to a recent report from the World Health Organization, the use of digital health technologies can improve women's health and promote equity by improving the access to healthcare services and enhancing maternal health through the provision of pregnancy support.^63^ For example, digital maternal health has shown positive impact in increasing the attendance and uptake of ANCs by providing maternal health education and supporting self-care management in LMICs.^64–68^ Although there has been a greater use of digital health in LATAM,^69^ its application in antenatal care has been limited and mainly on the use of the phone to support communication and less in monitoring pregnancy-related conditions.^39^

For example, text messages have been used to send general information to pregnant women on topics such as nutrition, diet, warning signs (e.g. hypertension), and motivational messages with the aim of reducing inequities related to access of maternal information in Peru.^70^ In addition, electronic health records (EHRs) have been implemented to support counseling and provide warnings and reminders to Peruvian healthcare providers about pregnant women missing appointments or cases requiring particular attention.^70^ In Argentina, a study investigated pregnant women's access and use of mobile phones and reported women's interest in using SMS and phone calls to receive prenatal care information.^71^ In Brazil, a study reported positive perceptions by pregnant women in validating a prenatal care app that supports the creation of birth plans and sharing them with others.^72^ In Mexico, a wireless monitoring device has been used in a rural clinic to support remote fetal monitoring for pregnant Indigenous Mayan women experiencing high-risk pregnancies.^17^ In addition, researchers have been analyzing medical records to facilitate the early and automatic diagnosis and treatment of urinary tract infections during pregnancy.^73,74^ However, little is known about the potential of digital health in antenatal care of high-risk pregnancies in LATAM.^17,39^

In Ecuador, a non-profit organization is promoting the prevention of mother-child transmission of HIV during pregnancy, childbirth, and breastfeeding through information on their website.^75^ The Ecuadorian MSP has created the nominal monitoring system (SISEN Salud), which monitors the delivery of services to pregnant women and allows healthcare personnel to generate alerts when they have not received vitamin supplements.^76^ In addition, a digital platform has been implemented to enable healthcare professionals, researchers, and community organizations to have access to data on pregnancies among girls and adolescents as a starting point to support the development of preventive strategies.^77^ Additional governmental initiatives include the use of tele-education for prenatal counseling delivered by specialists in Gynecology and Nursing,^78^ a mobile application (“Infancia con Futuro”) providing general pregnancy-related information,^79^ and another one (“Infancia-ec”) to support the monitoring the baby's development, receive reminders of prenatal appointments, and accessing general health recommendations.^80,81^

Many commercial apps are also available in the Apple and Google Play Stores to provide pregnancy information (e.g. nutrition, fetal development, physical activity)^82^ or facilitate self-monitoring (e.g. weight or bump tracking, contraction counter).^83^ However, many of these applications have been implemented for high-income countries with low acceptance among low-income and non-English-speaking women.^84^ Moreover, recent reviews of pregnancy apps have highlighted shortcomings including low quality and reliability of information, lack of clinical validity and regulation, and lack of relevance for local health contexts.^82–85^ For example, although 58.1% of the apps reported by Lazarevic et al.^83^ are accessible to pregnant women in Ecuador for download, they are mostly available in English and have not been designed for the Ecuadorean or LATAM contexts.

## Aim and methods

This qualitative study aimed to further understand the specific needs and practices of pregnant women and healthcare professionals while caring for pregnancy complications. We use a qualitative case study method^86^ to explore the contextual challenges when accessing and interacting with antenatal services, and explore opportunities for digital health in antenatal care in Ecuador, a particular country in LATAM where research in this area is still limited.^39,68^ We build on our previous work understanding pregnancy care practices and related care infrastructures in India that highlighted the need to further understand the specific experiences, needs, and challenges of pregnant women during complications.^9,10,87^ In addition, our cross-disciplinary reviews on the use of digital health for pregnancy and maternal health^42,68^ show limited work on digital maternal health in the Global South. Our study is situated within an interpretive research paradigm taking a practice-oriented research approach^88–90^ to provide an in-depth understanding of real-life contexts,^86,91^ and how sociomaterial factors influence pregnancy care^10,38^ and digital health practices^92–94^.

### Study design and research settings

Ecuador is a multi-ethnic and multicultural country with a total population of 16,938,986, comprised of 77.5% Mestizos, 7.7% Montubios, 7.7% Indigenous, 4.8% Afro-Ecuadorians, 2.21% White, 1.44% Mulatos, 1.33% Black, and 0.12% of other ethnic groups, according to the most recent census.^95^ The country is divided into four regions (the Coastal Lowlands, the Andean Highlands, the Amazon Rainforest, and the Galapagos Islands). It has 24 provinces. Our study was conducted in two of the most populated Ecuadorian cities: Guayaquil in Guayas province, located in Ecuador's coastal region, and Quito in the Pichincha province, located in Ecuador's highlands region. We also included the city of Riobamba, which is the major city in the Chimborazo province, located in Ecuador's central highlands, where 52.40% of the population resides in rural areas.^96^ The Pichincha and Chimborazo provinces have the largest indigenous population,^95^ and Chimborazo has the highest maternal mortality rate in Ecuador.^97^ The research team included a cross-cultural and interdisciplinary group of researchers (two from the UK and six from Ecuador) with backgrounds in social sciences applied to health, public health, human-computer interaction, health informatics, and data science. Local researchers have longstanding partnerships with healthcare services and local communities in Guayaquil, Quito, and Riobamba.

### Participant recruitment

We purposefully^86^ invited pregnant women (PW) with pregnancy complications who were attending antenatal consultations, as well as healthcare professionals (HCPs) who provide antenatal services and have experience managing pregnancy complications. To ensure diversity in terms of geographic location and health coverage, we recruited participants from three Ecuadorean cities, and across eleven healthcare institutions (10 urban and 1 rural) including three private hospitals, three public hospitals, and five health subcenters. In Guayaquil, we engaged with one of the largest private hospitals in Ecuador that specializes in women's health, a public hospital that is part of the IESS’ hospital network, and two MSP health subcenters: a type B and type C subcenter (see Figure 1). In Quito, we recruited participants from two public hospitals specializing in obstetrics and gynecology and part of the Ecuadorian MSP's hospital network. We also engaged with a private hospital and an MSP type A health subcenter in Puembo (rural area). In Riobamba, we engaged with a private hospital and two MSP type A subcenters. The research teams in each city were granted permission by the authorities of the healthcare institutions involved. We received two ethical approvals: one from the Ethics Committee (ref. CEISH-USFQ 2019-095M) from Universidad San Francisco de Quito in Ecuador, and one from University of Leicester Ethics Committee (ref. 20836-nxvd1-scen:informatics) in the UK. The research teams introduced themselves and explained the purpose of the study. Participants received a participant information sheet describing the study procedures, potential benefits, and data confidentiality. None of the researchers had previous relationships with the participants. Written informed consent was obtained from all participants prior to the studies.

We recognize expectations for sample size in qualitative research^98^ (e.g. 9–17 interviews or 4–8 focus groups^99,100^), but the concept of “data saturation” conflicts with our interpretivism research paradigm and the values of reflexive thematic analysis.^101,102^ Data saturation aligns more with (post)positivism approaches and other types of thematic analysis (coding reliability and codebook approaches),^102^ rather than embracing the situated, subjective, and reflexive engagement of the researcher in non-positivist qualitative research.^103^ For this reason, we followed principles of sufficiency^98^ and information power^104^ and the values of a non-positivist qualitative paradigm.^101^ Rather than anticipating a sample size,^102,105^ research teams agreed that our data (33 interviews with HCPs and 8 focus groups with PW with complications across three cities) were extensive and collectively provided us with a rich understanding of the socio-technical challenges and opportunities for digital health regarding women's access to and interactions with antenatal services during pregnancy complications, while acknowledging the potential for new insights.^102,106^

We also acknowledge the shortcomings of existing checklists for reporting qualitative research (e.g. COREQ^107^) that are suitable for quantitative/(post)positivism approaches and not applicable to all types of qualitative research, especially for non-positivist qualitative research.^108^ In addition to saturation, member-checking, coding trees, and triangulation are (post)positivist-friendly concepts that are inappropriate and irrelevant for non-positivist qualitative research where subjectivity is an inherent part of knowledge creation.^101,109^

### Data collection

The manager or director of each institution helped us identify the participants, and we conducted in-person interviews and focus groups between August and September 2019. Table 1 shows an overview of the methods per research setting.

**Table 1.** Description of data collection methods^a^

				Guayaquil		
		Interviews (INT)		Focus Groups (FG)		
Healthcare service	Qty	Qty	INT codes	Qty	FG participants (FGP)	FGP codes
Public Hospital (PH)	1	4	PH, HCP1–4	–	–	–
Private Hospital (PVH)	1	3	PVH, HCP1–3	2	8	PVH, PW1–8
Public Health Subcenters (PHS)	2	6	PHS, HCP1–6	2	9	PHS, PW1–9

				Quito		
		Interviews (INT)		Focus Groups (FG)		
Healthcare service	Qty	Qty	INT codes	Qty	FG participants (FGP)	FGP codes
Public Hospital (PH)	2	2	PH, HCP5–6	1	9	PH, PW1–9
Private Hospital (PVH)	1	2	PVH, HCP4–5	–	–	–
Public Health Subcenters (PHS)	1	1	PHS, HCP7	–	–	–

				Riobamba		
		Interviews (INT)		Focus Groups (FG)		
Healthcare service	Qty	Qty	INT codes	Qty	FG participants (FGP)	FGP codes
Public Hospital (PH)	–	–	–	–	–	–
Private Hospital (PVH)	1	5	PVH, HCP6–10	1	6	PVH, PW9–14
Public Health Subcenters (PHS)	2	10	PHS, HCP8–17	2	11	PHS, PW10–20
Total 11		33		8 43		

^a^All interviews with healthcare professionals start with the code HCP. All focus groups with pregnant women start with the code PW.

#### Interviews with healthcare professionals

We conducted 33 semi-structured interviews with healthcare professionals providing antenatal services at different levels of the healthcare infrastructure: public and private hospitals and health subcenters. Our key informants included nurses, family doctors, obstetricians, gynecologists, and obstetrician-gynecologist doctors. Interviews took place at the healthcare institutions and lasted between 15 and 57 minutes and covered HCPs responsibilities regarding caring for pregnant women with complications, their workload, and the emotional support received at work. We asked about existing barriers in the care of pregnant women with complications, their use of technology to support their work, and ideas for technology to improve their work and the care of women with pregnancy complications.

Details of the interviews are available in Supplementary Materials. Interview questions were pilot-tested through eight preliminary interviews in Riobamba, which were excluded from the analysis. Table 2 shows an overview of our 33 HCPs participants per city, including nurses (*n* = 9), family doctors (*n* = 9), obstetricians (*n* = 3), gynecologists (*n* = 7), and obstetrician-gynecologists (*n* = 5). They have treated pregnancy complications such as pre-eclampsia, gestational diabetes, vaginal bleeding, placental complications, obesity, etc.

**Table 2.** Characteristics of healthcare professionals.

		Guayaquil		Quito		Riobamba	
Specialist	Higher education (years)	n	AvgExp (years)	n	AvgExp (years)	n	AvgExp (years)
Nurses	4	5	5.8	2	5.5	2	8.0
Family Doctors	9	1	8.0	2	7.5	6	4.0
Obstetricians	4	1	26.0	1	10.0	1	5.0
Gynecologists	10	2	29.0	–	–	5	11.0
Obstetrician–	10	4	23.2	–	–	1	4.0
Gynecologists							
Total		13		5		15	

#### Focus groups with pregnant women with complications

We engaged a total of 43 pregnant women with complications through eight in-person focus group discussions across different care settings and cities. Upon the recommendation of local researchers and healthcare professionals, focus groups were chosen as they provide a rich understanding of participants’ experiences,^110^ deeper insights,^111^ and are particularly suitable for health research^112^ especially around sensitive topics^113^ on women's health.^114^ Focus groups can make women feel more comfortable sharing experiences, relying on their “collective sense of women's solidarity.”^114^ Focus groups took place at the different healthcare institutions and ranged in size from three to nine participants and lasted between 15 and 75 minutes. The inclusion criteria were: (a) to be at least 20 weeks’ gestation, (b) to have pregnancy complications (e.g. diabetes, pre-eclampsia, anemia, malnutrition, etc.), (c) to be between 18 and 45 years old, and (d) to have attended at least one antenatal appointment. After explaining the purpose of the study, discussions centered around their opinions, experiences, and challenges while interacting with antenatal services, their information-seeking practices and concerns, their use of technology, and their perceptions of how technology can help them manage their complications. Details of the focus group questions are available in Supplementary Materials. Our focus group guide was pilot-tested in a feedback session with a medical doctor. A researcher moderated discussions and encouraged participants to talk freely and ask questions at any time, keeping a calm and trusting environment. Table 3 shows basic socio-demographic information of our 43 PW participants. Pregnant women were between 18 and 42 years old. The majority attended school between the elementary and high school levels (*n* = 31), while the remaining participants obtained some form of higher education (*n* = 12). All PW participants have accessed at least one public or private healthcare facility during their current pregnancy to treat their complications.

**Table 3.** Characteristics of pregnant women with complications recruited.

	Guayaquil			Quito			Riobamba		
Age	Min	Max	Avg	Min	Max	Avg	Min	Max	Avg
Age of PW (years)	20	42	29.29	19	39	27.90	18	40	26.80

Education	*n*		%	*n*		%	*n*		%
PW with partial or completed Elementary	5		29.41	1		11.10	11		64.71
PW with partial or completed High School	8		47.06	2		22.20	4		23.53
PW with partial or completed Higher Education	4		23.53	6		66.70	2		11.76
Total	17			9			17		

Each research site had different practices regarding compensation for research participants. Participants from the health subcenters in Guayaquil were compensated for transportation. In Riobamba, the participants from the health subcenters received a meal. The remaining participants did not receive any compensation upon recommendation of the local researchers as it was considered inappropriate.^115^

### Data analysis

We conducted all interviews and focus groups in Spanish, which were audio-recorded with the consent of the participants and anonymized for analysis. The three research teams from Quito, Guayaquil, and Riobamba transcribed the recordings independently for a multi-stage inductive thematic analysis. Initially, one member of each local team familiarized with their data and thematically analyzed their data^116,117^ by identifying codes, refining them, sorting them, and grouping related codes into themes to maintain the contextual nuances of each setting. The research team in Guayaquil used the NVivo software to support the analysis, while the other two teams did not use a particular software as it was done manually with the support of a Word processor.

As our goal was to identify the contextual challenges that pregnant women and healthcare professionals faced while interacting and accessing ANCs, Braun and Clarke's reflexive thematic analysis approach was deemed the most suitable,^116,117^ in contrast to other approaches that prioritize the quantification of patterns and accuracy, for example coding reliability approaches, qualitative content analysis, etc., aligned to (post)positivist research values.^118^ Preliminary results from the three cities were shared in a research workshop to review initial themes and support collaborative analysis to develop themes further. Then, three researchers performed a second round of analysis focusing on “constraints” and integrating the findings across the research sites to generate a bigger picture of the challenges pregnant women and healthcare professionals face while accessing or providing antenatal services. The researchers met regularly to discuss the overarching themes, shared the combined themes for comments with the interdisciplinary team, and consolidated the themes upon the final writing of this article.

## Results

The analysis revealed three overarching themes: (a) Constraints in the Access and Structuring of Antenatal Care, (b) Unintended Consequences, and (c) Perceived Value in Technology for Pregnancy Care. These major themes and their corresponding sub-themes identified in the analysis are presented below.

### Constraints in the access and structuring of antenatal care

#### Material constraints

The materiality of healthcare environments enabled and constrained the provision of antenatal care. One of the major context-specific challenges relates to the physical infrastructure of public hospitals and MSP health subcenters, which is often not optimal and lacks the necessary resources to provide antenatal care. A way to deal with these material constraints is to visit other healthcare facilities for specific examinations not available in public services. For example, a pregnant woman attending a private hospital shared with us her previous experiences at the public health center by stating “*In the [public] health center there is no ultrasound machine, so they should fix the machine or buy one to do an ultrasound because there is no…*” (PVH, PW8). Indeed, a healthcare professional from a public hospital confirmed the need “*to upgrade diagnostic equipment that is already out of date, ultrasound machines that have already passed their expected lifespan a long time ago*” (PH, HCP3). Besides medical equipment, public hospitals often do not have enough medical supplies to provide good-quality antenatal care. A healthcare professional noted:

“Sometimes we have a shortage of supplies, a shortage of medicines. . . for example, there are tests that we do not do at the hospital, and we have to do elsewhere, take the patients out of the hospital, do the exams, and bring them back to the hospital.” (PH, HCP6)

The situation is even more problematic at public health subcenters, as we observed they only have basic instrumentation (e.g. Pinard stethoscopes, blood pressure monitors, etc.) for pregnancy care. Some health subcenters do not have laboratories, pharmacies, additional equipment, or medical supplies needed for hospitalization, case monitoring, or emergency care. For example, a health professional stated: “*Due to our level of care, we do not have our laboratory, and we cannot perform basic or specific examinations. We must refer patients to other places, and this bothers them*” (PHS, HCP12). Indeed, another healthcare professional told us “*this is a type A unit [public subcenter], where we should not have emergencies because we do not have the medications nor the supplies to treat emergencies*” (PHS, HCP7).

#### Spatial constraints

Antenatal care is usually hampered by spatial constraints, especially in public health subcenters, as healthcare facilities do not have adequate space to provide appropriate support to pregnant women. The small consultation and waiting rooms impact patient privacy and overall care delivery. A healthcare professional commented:

“It [public health subcenter] is very small. We do not have enough infrastructure to accommodate many people. We need private offices for doctors because you listen to them and can even observe other consultations. They [MSP's managers] told us that they are going to give us a new building, but it's been two years, and they don't give us anything.” (PHS, HCP10)

The hygiene aspects of private healthcare facilities were associated with a positive experience, especially in hospital environments. A pregnant woman commented:

“From the moment you arrive at a private hospital… you can see that the physical space is clean and very well presented. That gives you confidence for care. The place where I receive treatment is good because you can see the doctor's commitment to the patients.” (PVH, PW9)

However, hygiene practices were a major issue in public health centers. A participant shared her negative experiences:

“The bathrooms, that day, I was going to have a urine test because I didn't urinate in the house as I didn't feel like urinating, and when I went to the bathroom [at the health center], the bathroom was already too dirty, I don't know if it was because they didn't clean it, but the floor looked very dirty, and the whole bathroom was like this.” (PHS, PW4)

#### Technical constraints

The materiality of information technologies and other information artifacts also enabled and constrained the provision of antenatal care. In particular, public health subcenters of the MSP use an electronic health record system called Health Care Registry Platform (Plataforma de Registro de Atención en Salud—PRAS) that enables a comprehensive registration (e.g. tests, symptoms, diagnosis, treatments) and web-based access to patient data. The public hospitals and health centers of the IESS have a different electronic health record system called MIS-AS/400 to access medical history, visualize previous diagnoses, manage medical appointments, etc. Sometimes, these systems fail; for example, a healthcare professional from a public hospital stated that the MIS-AS/400 system failed with *“a frequency of 2 or 3 times a year”* (PH, HCP3). The situation at the public subcenters was worrisome as the PRAS system failed more often due to several reasons, as explained by a healthcare professional, *“many times it [PRAS] has failed, when the electricity or the Internet drops out, in those occasions, we have to make the medical history manually, and when the system comes back online, we have to update the information [in the PRAS]”* (PHS, HCP11). In addition, some healthcare professionals perceived that their computer systems become very slow during the morning due to the excessive web requests from different health centers of the public health network.

“At this time [in the mornings], for example, the [PRAS] system is very, very slow. Yes, it is when we have already entered all the data. Imagine, we are many health subcenters in this district that enter and enter. . . [information]. I can imagine, I don't really know, I don't know much about computing, but at this time [in the mornings] it gets saturated….” (PHS, HCP5)

In addition, healthcare professionals commented on the lack of integration of EHRs from different public health providers (MSP and IESS) and other public and private institutions. A healthcare professional from a public healthcare center commented, *“I think that all [electronic health record systems] should be unified. We should have only one system, which may be good and fast”* (PHS, HCP4). Similarly, EHRs in private hospitals do not integrate with any system from the public health network.

“We are only in a network with Junta de Beneficencia hospitals [private hospitals]. For example, if a patient is transferred from the IESS [public hospital], I do not have access to the tests that have already been done or to the care that has been done in another IESS hospital.” (PVH, HCP3)

#### Organizational constraints

The use of antenatal care is also affected by organizational constraints as the rules and processes of healthcare services challenge the development of an optimal patient–doctor relationship and the responses to any complication. For example, pregnant women mentioned the difficulty of getting an appointment at the public health subcenters. Women must insist several times calling to get an appointment as stated by one participant, *“I phoned 171 times to make an appointment, for this appointment I was calling almost for seven days because there were no time slots in the agenda. I was scared…”* (PHS, PW4).

Pregnant women also expressed dissatisfaction with the attitudes of healthcare professionals from public health institutions, especially after experiencing a high level of care in private institutions. With respect to healthcare professionals from private hospitals, participants commented that *“from the moment you enter, they treat you with kindness”* (PVH, PW9), and *“… are always monitoring us, controlling our [blood] pressure, it has been excellent care”* (PVH, PW5). This sometimes does not happen in public health institutions due to other organizational constraints (e.g. administrative arrangements, time of consultation, availability of doctors, etc.). Indeed, HCPs acknowledge pregnant women's dissatisfaction.

“There are complaints… there is not adequate care because it [healthcare service] collapses, it is impossible for a doctor to see 30 patients, [or] 80 patients in 24 h a day, it is impossible.” (PH, HCP3)

In addition, pregnant women highlighted the lack of empathy skills in healthcare professionals’ daily practice, especially at the healthcare subcenters. A pregnant woman commented that *“some [HCPs from public health subcenters] care well, others are rude”* (PHS, PW11), and another participant expressed:

“Sometimes, we [pregnant women] go back [to doctor's office] to ask something else, and they [HCPs] look at us badly, like saying, why do we go back?.” (PHS, PW10)

#### Other everyday life constraints

The socio-material practices of everyday life outside healthcare settings also imposed some constraints on accessing antenatal care. For example, additional reasons that healthcare professionals received from patients for not attending antenatal care check- ups include (a) living far from the health subcenter and (b) having family or work responsibilities. An HCP commented:

“From my perspective, many patients do not have control of their cycles. . . Sometimes, the situation becomes more complex as they live far from a health subcenter or have many children they cannot leave alone. There are various situations that patients tell us that they face. . . their workload and family burden so they cannot attend regular antenatal checkups.” (PHS, HCP5)

### Unintended consequences

#### Additional data work, duplication of information, and incomplete records

As presented above in the technical constraints, the PRAS system often has service failures, forcing health professionals to temporarily use paper records, increasing their workload as they need to do paper-based registrations until the digital system is back online. In addition, the lack of data integration among different healthcare provider systems causes a duplication of patient information. A healthcare professional stated:

“It would be good if the zone 8 program for pregnant women [public hospital system] and the PRAS system [health subcenter system] were unified so that we do not perform double work, which is also prone to errors. Then imagine collecting all the data the patient gave me again… that can cause me to forget something to register because I no longer remember….” (PHS, HCP3)

Indeed, missing information in the electronic health record influences the utilization of antenatal care, especially in subcenters, *“I used to go for the [antenatal] checkups to the subcenter, but they had me from side to side, and they lost my clinical record … then it bothered me a bit”* (PH, PW6).

But the extra work and the high risk of missing information are not the only consequences, as stated by a healthcare professional from a public hospital, *“When the system [MIS-AS/400] crashes, it creates chaos, consultations are delayed, patients complain as they were scheduled at that time or an hour has passed, and they still have not been seen…. How can we attend to them without the system working?”* (PH, HCP3). At the healthcare subcenters, delays can indeed impact pregnant women's experiences, the scheduled appointments, and the workflow of patient care because of organizational constraints:

“It is a difficulty for us since we have to adjust the time with the other patients, but this under no circumstances limits the time of care for the pregnant woman. We have a time of care for the patients of 15 min. We take 40 min to an hour with a pregnant woman, but they are given that time, and then we have to see how we give time to the other patient.” (PHS, HCP16)

#### High uncertainties and delayed diagnosis in a fragmented system

As a result of the organizational constraints, one of the main reasons for the low utilization of antenatal services is the late recognition of pregnancy, as some pregnant women reported not knowing they were pregnant for weeks, where late recognition could lead to delayed responses to complications.

“Until I was eight weeks pregnant, I was working normally, but when I went to the doctor, I did not know that I was pregnant. They [HCPs] had to hospitalize me for five days because I had a threatened abortion, due to a strong infection, for this I had to quit my job and just rest lying down.” (PVH, PW10)

In public institutions, health professionals make diagnoses of pregnancy complications and determine which treatment women should follow or make referrals to specialized or private hospitals. However, the reality is very complex. Several participants expressed their discomfort with the health subcenters. They considered their medical attention deficient because their pregnancy complications were not diagnosed on time.

“I previously attended my last checkup at the [public] hospital in Yaguachi, where I live. There I had a blood pressure of 150 over 90, and the doctor diagnosed pre-eclampsia without doing any medical test or anything and I was hospitalized there [at public hospital]. Then, they [HCPs] referred me here [private hospital in the city]. They [HCPs] did medical tests, ultrasounds, analyses, and a urine test to see if I had proteinuria. The results were negative; then it was only the blood pressure…. They [HCPs] discovered that it was not pre-eclampsia but gestational hypertension, because I don't have any other symptoms… with the medication, they controlled my blood pressure, and I got a normal blood pressure now.” (PVH, PW1)

Another pregnant woman shared with us the issues she faced at the public health center that delayed the diagnosis of her child's condition, which was later confirmed by an additional test initiated by herself.

“In the [public] health center, there were many examinations that they did not perform… and they [HCPs] never gave me a diagnosis for my child. It was through a private ultrasound that the child was diagnosed with that [hydrocephalus].” (PVH, PW5)

#### The need for emotional support for HCPs

The physical and organizational constraints also influence how healthcare professionals feel as they perceive their work is undervalued by their patients in public health institutions. One HCP noted:

“Our work is not always valued because pregnant women, by policy, must be attended for an hour, but this is not understood by the other patients who are waiting. For example, pregnant women sometimes come without having breakfast, and they urinate every moment [delaying the consultation]. Due to the lack of infrastructure and personnel, there is no way to attend to them quickly.” (PHS, HCP8)

In addition, healthcare professionals from public and private healthcare institutions stated that they can handle high levels of stress, however, they lack emotional support. A healthcare professional expressed:

“We all handle a fairly high level of stress, right? Because of the many responsibilities we have on our shoulders, it is good to look for a professional knowledgeable in the matter with whom one can open up very widely and unload all those tensions that one can carry, and that cause disease. For example, I am hypertensive; it started one year and five months ago, so we do need emotional support, especially if it is given by a professional.” (PHS, HCP3)

Moreover, healthcare professionals’ busy workloads do influence their well-being in many ways, as caring for high-risk pregnancies is an emotionally demanding job function and can bring legal liability issues. A healthcare professional commented:

“We do not have any emotional support, but it is very necessary because, as I mentioned, there have been several maternal deaths, and it is the stress when one comes to work, and they [colleagues] say that such a patient died, immediately I am going to look in the planning if it is my patient. They [lawyers] from Quito can start the lawsuits with the families [against HCPs]. It is very stressful because we leave the patients well, and they get worse because they [patients] might have ignored the instructions.” (PHS, HCP10)

#### Emotional and physical work that women and families do to help managing complications

Navigating the physical and organizational constraints and the uncertainty about their condition was challenging for pregnant women who also need to manage their worries and fears. A participant commented:

“At first, I was worried because the pressure would not go down anyway, and little by little, it was normalizing, and I was afraid that they would take the baby out early and that it would come out prematurely and that stuff. I’m afraid of getting pregnant again because of being hospitalized….” (PVH, PW1)

To deal with all these concerns, other family members (e.g. parents) can offer care and support to help women manage their complications in everyday life. A pregnant woman shared a positive experience of this practice.

“I now live with my parents, with mom and dad, they are always looking out for me… My mother sleeps with me and always knocks to check on me…My husband supports me a lot, but he is far away; he does not share the day-to-day of my pregnancy, but my parents do, and this has brought us closer together…. (PVH, PW11)

However, the lack of contact with their immediate family for a long period of time can increase the emotional distress of pregnant women. The same participant commented:

“… I feel depressed because my family [husband and son] lives an hour and a half away. Now I can't travel every day. I need my husband, and since I am not with him and my first child, it depresses me a lot; apart from my symptoms, I cry a lot….” (PVH, PW11)

In other cases, pregnant women can feel isolated even while living with their husbands. A participant mentioned:

“I stopped visiting my family because I live with my mother-in-law and my husband. My ex-husband has my two children right now because I can't strain and take them to school… I can't do anything because I have to rest… I don't go out alone; I only go when I have a consultation.” (PHS, PW1)

The lack of attention to women's emotional well-being during pregnancy can have profound long-term consequences that can even affect the relationship between the mother and the newborn.

“Psychologically, I have gotten sick; I get cranky by the baby because it hurts a lot; when I walk, when I take a rest, my belly hurts. The doctors tell me to hold on and take the vitamins that it is a high-risk pregnancy. I have lost a lot of weight. I am not hungry or sleepy. It is very different from the first pregnancy. They [HCPs] keep doing tests on me, but they do not give me a diagnosis as to why I have so much headache and bellyache. It does not calm the itch. Psychologically, I do not want the baby to be a nuisance. I hate the baby….” (PVH, PW11)

### Perceived value in technology for pregnancy care

#### The potential of digital, personalized, and trustable information

Healthcare professionals talked about the potential digitization of the maternal booklet, an information-carrying object pregnant women receive during their first antenatal consultation to enhance information access. An HCP stated:

“We have a physical booklet given to them [pregnant women] in the first antenatal checkup. It shows all the risk factors and complications that can occur per quarter, and this should be digital because who does not have a smartphone.” (PHS, HCP10)

However, healthcare professionals also suggested being cautious with the level of information that is given to pregnant women as technology can be a “double-edged sword.” It can increase access to information but can also cause anxiety and stress in pregnant women. A healthcare professional highlighted these issues, the need for trustable information, and the potential benefits of digitizing the maternal booklet to support self-care practices.

“I see that [technology] as a double-edged sword, well… Most of them [pregnant women] have cell phones… not everything is true… and suddenly if a woman, like many do, seeks information but she does it in an application or in a place that is not reliable… she enters the consultation very worried because she read, [or] saw… [or] is very distressed regarding a situation that in reality differs from her interpretation… for education we provide the ’carnet’ [maternal booklet]… that contains the calendar of pregnancy [prenatal appointments]… many topics are included, it is not only about physical activity but we also talk [use it during consultation]… to get to know the concerns they have, also in some way we try to motivate them [to use it]… let's say… a pregnant woman has to take a test… but the next appointment is in 3 weeks, so the pregnant woman can take advantage of that [maternal booklet]… [and say] “Doctor, I have this exam…” then it seems to me that this technological part for self-care is a situation that can be upsetting… it can help if it is supervised by a professional.” (PHS, HCP3)

Regarding the digital version of the maternal booklet, pregnant women also expressed the need for personalized information that can be provided by trimesters to make them understand if everything is fine or help them recognize any warning signs of complications. A pregnant woman participant commented:

“As the pregnancy goes by trimesters, there may be a risk, for us to be prepared, and it would be important that there is a reminder of what could happen, a guideline to be alert, to know what is not normal.” (PVH, PW11)

Pregnant women also mentioned the potential use of videos to convey pregnancy information, especially to young people, to avoid unintended pregnancies or complications. They also expressed the desire for this information to come from health centers. A participant commented:

“I would like video applications, food applications, how to prevent infections, there are so many bacteria during pregnancy; and also, an application for the youth, since there are so many young girls who are getting pregnant; and also, through social networks, to make a video, something like that, maybe young girls can be better informed… and also the Internet could help, there are also many young people who do not receive help from their parents… I also hope health centers publish videos and other information on Facebook or YouTube… then I could look for information that sometimes I do not know, or that they do not want to explain, then I could search there.” (PHS, PW3)

#### Facilitating ad hoc communication and information sharing

Participants perceived value in technology that could facilitate ad hoc communication and information sharing between healthcare professionals and pregnant women. Participants regarded information coming from HCPs as valid and important. A participant commented:

“Any information that is received through the phone from a doctor is very important, either video, chat or video call, whatever, as long as it is useful for us who are pregnant. I would like information about pregnancy risks, care, food… everything is very important and is a support for us” (PHS, PW4).

Another participant commented on how an open chat could help establish a communication channel outside the healthcare settings to receive information.

“It would be more comfortable for us to have this type of information [care for high-risk pregnancy] in an open chat, to inform ourselves with the doctor, and we would be maintaining communication with the doctor once outside the hospital.” (PVH, PW2)

Indeed, healthcare professionals have suggested contacting them in case of doubts, and *“they [PW] are constantly calling me and asking questions via WhatsApp”* (PH, HCP4). In addition, HCPs expressed value in using chats like WhatsApp to support inter-institutional communication as a workaround considering the fragmentation of healthcare services, for example between hospitals and health subcenters to share information, communicate emergencies within hospitals, or facilitate patient transfers from health subcenters to third-level hospitals. A health professional shared:

“We do not have a dedicated ambulance as a health subcenter, but [if something happens] we have to activate a code. Red code when it is an emergency as such, we upload the case through WhatsApp using a message, and they [hospital staff] authorize the transfer of the patient … all the doctors from the different operating units, the district director, the hospital director and the emergency physicians that we have in the hospital are members in these WhatsApp chat groups.” (PHS, HCP7)

HCPs also mentioned using WhatsApp to support information sharing and interdepartmental communication. A healthcare professional commented *“we also have a general chat [in WhatsApp] for all the area managers to keep everyone informed”* (PH, HCP1). WhatsApp is also used for phone calls in case of emergencies in the public health subcenters of Guayaquil, *“as we [HCPs] need to act immediately.”* (PHS, HCP4)

#### Supporting the integration of heterogeneous care systems

Healthcare professionals also proposed ideas around improving the integration and coordination of EHRs across different institutions and the potential benefits of having the patient's history accessible. A healthcare professional expressed:

“For example, when I type Juanita Pérez [fictitious patient's name], the system should show me that she lives in Machala [city located in a different province] and all the health services the patient has received. So if she [pregnant woman] accessed healthcare services through the IESS, which includes the Rural Social Security, the Ministry of Public Health. . ., or whatever, you can open using the patient's name or type the identification number [national ID] so that all the [antenatal] checkups and medical history are shown regardless of the institution, and what [procedures, treatments, etc.] have been done. So, when we receive a patient, we would know the patient's medical history and save a lot of time and even resources.” (PHS, HCP4)

## Discussion

### Summary of findings

Our findings elucidate the socio-technical challenges in accessing antenatal services during pregnancy complications and the opportunities for digital health from different healthcare institutions in three major cities of Ecuador.

The first key finding relates to the constraints in the Access and Structuring of ANCs that provide a contextual understanding of the existing material, spatial, technical, organizational, and everyday life constraints that influence antenatal care in Ecuador. Material constraints show how public hospitals and health subcenters often lack essential resources, such as diagnostic equipment, medical supplies, laboratories, and pharmacies, making pregnant women with complications to seek services elsewhere. Spatial constraints particularly impact public health subcenters that have inadequate space for consultations and waiting areas, compromising patient privacy and healthcare delivery. Hygiene issues in public facilities also negatively affected women's experiences attending antenatal services. Technical constraints show how electronic health record systems failed due to power outages, internet issues, or system overloads. Organizational constraints highlight how pregnant women encountered difficulties in booking appointments, faced long waiting times, and experienced dissatisfaction with the attitudes of healthcare professionals in public institutions. Overburdened healthcare professionals struggle to provide adequate care due to high patient loads and limited resources. Everyday life constraints, such as long distances to health centers, family responsibilities, and busy work schedules, preventing pregnant women from attending regular antenatal checkups. These constraints collectively hinder the accessibility, quality, and efficiency of ANCs in Ecuador. Our findings expand previous research reporting socio-technical challenges and constraints in the access and utilization of ANCs in other LMICs (e.g. India, South Africa, Uganda, Pakistan).^11,41,119–121^

The second key finding relates to the Unintended Consequences (subthemes: additional data work, duplication of information, and incomplete records; high uncertainties and delayed diagnosis in a fragmented system; the need for emotional support for HCPs; and emotional and physical work that women and families do to help managing complications) of the aforementioned constraints that negatively impacted women's access and experiences of care and the provision of antenatal services for pregnancy complications. For instance, failures in electronic health record systems force healthcare professionals to temporarily rely on paper records, which increased their workload. The lack of integration among systems led to duplication of patient information and increased risks of missing data. On the one hand, incomplete medical records, combined with fragmented healthcare systems, resulted in delays in the diagnosis and treatment of pregnancy complications, leading to frustration and dissatisfaction among pregnant women. On the other hand, delayed recognition of pregnancy and delayed responses to complications increased the emotional distress for pregnant women, who often experienced uncertainty about their condition and treatment. Healthcare professionals also experienced stress due to heavy workloads, insufficient emotional support, and concerns about legal liability, particularly in cases involving maternal deaths. In addition, pregnant women and their families experienced emotional distress and isolation while coping with complications. The absence of family support and contact intensified their struggles, often resulting in negative feelings towards their pregnancies. Although our study was conducted before the COVID-19 pandemic, our findings highlighted how the lack of physical infrastructure, the unavailability of technical systems, the limited time with HCPs, and the lack of empathy of HCPs posed several socio-technical challenges to women's antenatal care that were exacerbated during the pandemic as shown by other studies.^122,123^ In fact, the pandemic made the unmet demands of the healthcare system more visible, as many antenatal care facilities closed, affecting the provision and utilization of maternal health services in Ecuador.^122^ The fragmented nature of ANCs, requires pregnant women to navigate between public and private services, increasing the burden on women and healthcare professionals. This fragmentation continues to persist after the pandemic and particularly impacting the emotional well-being of pregnant women with complications and their healthcare providers.

Our third main finding relates to the Perceived Value in Technology for Pregnancy Care that highlights the potential benefits of digital health in supporting pregnancy care in Ecuador. In contrast to a study with 640 Ecuadorian physicians that reported high use of Information and Communication Technologies (ICTs) in their practices,^124^ we found that very little technology was used. Aligned with a recent study of digital maternal health in Latin America countries,^39^ pregnant women and HCPs perceived certain value in technology to support and enhance pregnancy care experiences during complications. For example, healthcare professionals suggested digitizing the maternal booklet given during antenatal checkups to offer reliable, personalized information about pregnancy risks, complications, and self-care practices. This could help pregnant women recognize warning signs and manage their health more effectively. Both healthcare professionals and pregnant women emphasized the importance of accessing accurate and validated information to avoid stress from unreliable online sources. In addition, videos and apps from health centers were recommended as tools to educate pregnant women and prevent complications. Pregnant women expressed interest in using technology, such as open chats and video calls, to communicate with healthcare professionals outside of consultations. Healthcare professionals also used WhatsApp for inter-institutional communication and patient emergencies. They proposed integrating EHRs across institutions to streamline access to patient history, reduce duplication of work, and enhance coordination among healthcare providers.

Next, we discuss our findings highlighting opportunities for digital health in pregnancy care in Ecuador and beyond.

### The potential of digital health and equity in pregnancy care

Digital health has the potential to address inequities in maternal healthcare across urban and rural settings. Capasso and colleagues found that numerous facilities across Latin America and the Caribbean implemented some form of Information and Communication Technologies (ICTs) in maternity care and considered them as important tools to improve health access and care for pregnant women.^39^ Other tools, such as using AI machine learning algorithms, can examine data from various sources, including social media and patient records, to identify postpartum women at risk of mental health issues. These AI-driven tools aim to make mental health support more accessible and convenient. This continuous monitoring can ease the burden on healthcare facilities, particularly in remote and underserved regions with limited access to quality prenatal care.^125^ Telehealth has helped close care gaps for marginalized groups, including pregnant women with limited access to rural maternity services. It has contributed to the reduction of adverse intrapartum and postpartum outcomes. Teleconsultations via video or audio conferencing platforms have enhanced access to maternal-fetal care in rural areas.^126^

There is limited evidence on the cost and effectiveness of digital tools in healthcare. A systematic review found that digital health interventions can improve cost-effectiveness, benefiting both costs and health outcomes. Specifically, studies involving new mobile apps or web portal interventions showed particularly positive results. In that systematic review, half of the studies indicate that digital health interventions improved patient outcomes and efficiency, optimized human and technological resources, and consistently reduced healthcare service costs. However, comparing interventions remains challenging due to differences in study methods, cost perspectives, research contexts, and diseases.^127^ In Bangladesh, a cost-effectiveness analysis on a digital health intervention on pregnancy was estimated; the primary factors driving cost-effectiveness were the number of lives saved during child delivery at both facility and community levels, followed by program costs and population coverage within the mCARE system. It showed that in urban settings with high health system capacity and care-seeking for facility-based deliveries, lower program costs (due to high population density), and lower population coverage, cost-effectiveness can be as low as $172 per DALY averted. In contrast, it could be as high as $1287 per DALY averted in rural or hard-to-reach areas. This suggests that while the mCARE program can increase population and service coverage, it may result in suboptimal cost-effectiveness and higher societal costs compared to the status quo, unless the increase in care seeking translates into more lives saved through high-quality services, particularly during child delivery.^128^

Another systematic review included eight studies that assessed the cost-effectiveness of mHealth interventions to support women during pregnancy. All studies covered both high-income and low-to-middle-income countries. The interventions discussed in the studies were designed to improve maternal care, promote healthy weight gain during pregnancy, identify pregnancy risks, and help manage gestational diabetes. Four of the studies of moderate to high quality found that mHealth interventions, particularly those involving notifications, reminders, or personalized health education, were cost-effective. The remaining four studies reported that mHealth interventions were either inexpensive or cost-saving, though these studies were of low to moderate quality.^129^ A study made in rural India using a decision tree model based on local data evaluated the costs of implementing the mHealth intervention ReMiND in routine maternal and child health services. From a societal perspective, the ReMiND program was cost-saving, as it increased the preventive and consequently reduced illnesses during pregnancy, lowering the need for expensive treatments. Regarding health gains, ReMiND helped avoid 3.1 million maternal illnesses and 37,337 neonatal illnesses, saving 312 maternal lives and 150,000 neonatal lives.^130^ More high-quality research studies are needed to demonstrate the health benefits of mHealth interventions and evaluate whether they are a cost-effective use of resources, especially in LMICs.

### Supporting the invisible work managing infrastructural constraints in antenatal care

Our findings revealed several constraints that resulted in additional work, often invisible, for pregnant women and healthcare professionals trying to fix or work around these constraints to make things work for them.^131^ Although previous research has highlighted the importance of understanding the invisible work made by clinicians, patients, and non-clinical stakeholders,^132–138^ only a few studies focused on understanding the invisible work associated with pregnancy care^42,139^ while interacting with antenatal services during complications.^38,87^

In contrast to antenatal services in the Global North,^140^ the healthcare infrastructure in LMICs is fragmented and distributed, making the situation more complex,^9,43^ impacting antenatal care utilization.^141,142^ For example, in our study, the lack of medications required additional work by pregnant women and their caregivers trying to find medications in alternative pharmacies outside of the antenatal care settings due to the lack of medications within the healthcare system or the disconnection between healthcare services, going beyond taking medications.^139^

Furthermore, pregnant women not only need to find their medications by themselves elsewhere, but also need to deal with data integration constraints, for example bringing physical copies of health information from their referral and counter-referral processes. This highlights the amount of navigation^38^ and infrastructural^140^ work pregnant women do to realign multiple cross-organizational elements within the fragmented and distributed care infrastructure. Our findings suggest that digital health tools to support pregnancy complications should facilitate the navigation and infrastructural work of pregnant women and their families. In the Ecuadorean context, a mobile application using map-based visualizations to find the closest pharmacies^143^ can be used to support pregnant women and their families when a medication is needed to treat complications. In addition, mobile health records^144^ can be enhanced to facilitate the exchange of referral information while moving across different antenatal care institutions.

Aligned with Luton's study in Australia,^145^ our findings also revealed the importance of customized information for pregnancy care per trimester that can support women's navigation work^38,42^ and help them managing uncertainties during complications. However, this needs to be carefully considered as increasing women's awareness with unreliable information, as highlighted by healthcare professionals in our study, can also end in unintended consequences, such as causing stress and anxiety.^146^ To prevent an overload of incorrect information online, our HCPs participants suggested that a digital version of the maternal booklet pregnant women receive at the consultation can increase access to more reliable and personalized prenatal care information. In addition, our participants highlighted the need to understand what was normal during pregnancy and a digitized version of the maternal booklet can offer opportunities to visualize health parameters to support interpretation and self-reflection,^42,147^ identify early signs of complications and seek prompt medical assistance.^148–150^ Our findings suggest that digital health tools to support women with pregnancy complications should be designed to complement existing information-rich resources. The maternal booklet serves as an example, as this tool will increase access and visualization of validated information (e.g. pregnancy-related content, prenatal appointments, health parameters, etc.), thereby improving self-reflection, maternal education, self-care practices, and usage during consultations, particularly in the Ecuadorean context.

Aligned to previous research,^151,152^ our findings also point to different unexpected technical misalignments (e.g. service saturation) and breakdowns (e.g. lack of electricity) that have affected the availability and use of different systems that support antenatal services. Previous research has also shown how workarounds are often considered an act of compensation to fix problems and overcome infrastructural constraints that might involve additional work.^153–155^ In our study, healthcare professionals circumvented infrastructural constraints by, for example, performing non-routine articulation work,^156^ for example borrowing basic instrumentation from colleagues or by incorporating paper notes as a supplementary mechanism to temporarily record medical information until the EHR system was operational, or by using WhatsApp to support coordination and share information within and across healthcare institutions. The persistence of the paper system^152^ and handling the interdependencies between existing systems at different levels of the healthcare infrastructure highlight the amount of alignment work^157^ needed to negotiate how workarounds can be conducted within and beyond individual local systems, increasing their complexity.^153^ Our findings suggest that digital health tools to support pregnancy complications should be designed to enhance the integration, coordination, and alignment work between different antenatal care providers and their recipients. This will require an in-depth understanding of the socio-political context in each country, and the active participation of all relevant stakeholders.

In addition, careful consideration should be given to the unintended consequences of using WhatsApp, as healthcare professionals from eight public health institutions reported using it as a workaround in antenatal care. However, data shared in WhatsApp is not easily integrated into EHRs and could be vulnerable to potential privacy and security breaches in relation to sensitive health information,^158^ increasing legal and ethical concerns.^159^ To support the care continuum and coordination especially when the system fails, implementing a paper backup system to augment an electronic system^160^ with the same layout of the EHR system could be useful to facilitate the manual re-entry of patient information. However, this workaround does not significantly reduce the workload for healthcare professionals, as they must still manually re-enter patient data.^161^ Moreover, data connectivity can be unreliable in low-resource settings of LMICs^39,162–164^ and asynchronous mechanisms (offline data entry) could be implemented on EHR systems with features to automatically download and store patient data on local servers until it can eventually connect to the remote server^162–164^ to facilitate coordination^165^ and prevent worsening inequalities.^166^

### Enhancing the emotional well-being of pregnant women and healthcare providers

Research on mental health in LMICs is very limited.^167^ While recent research has examined the role of the human infrastructure in providing mental health support in India,^168^ investigating the socio-technical factors surrounding mental distress in LATAM contexts remains unexplored. In our study, Ecuadorian participants with high-risk pregnancies showed emotional distress due to the lack of contact with their families, the high uncertainties in managing their conditions, the possibility of losing their babies, etc. At the time of writing this paper, maternal mental health screening is not formally part of ANCs in Ecuador. However, if some mental health issues are uncovered, a referral will be made. In the Ecuadorean context, our findings suggest to integrate maternal mental health into existing antenatal services^169^ and to implement digital strategies for the prevention, screening, and treatment of physiological distress and mental disorders in pregnant women,^170^ especially during complications. This will help promoting more equitable antenatal services that can enhance parents’ emotional well-being as suggested by a recent study in South Africa.^171^

Our study shows how pregnant women and healthcare professionals perceived the value of digital health tools (e.g. videos, WhatsApp). Pregnant women can benefit from receiving customized information to support their self-care practices and enhance health equity,^70^ but also to share information with others and especially with healthcare professionals, promoting trust and empathy.^172^ Sharing the self-reporting of psychological information during pregnancy can enhance treatment and care^173,174^ and foster good therapeutic relationships between healthcare professionals and pregnant women,^172^ which is key to promoting women's emotional well-being.^175^ While healthcare professionals mentioned the use of WhatsApp to communicate with pregnant women outside the consultation, careful attention should be given to the level of intrusiveness of this App^87^ as receiving calls or messages outside working hours can also increase the stress in healthcare professionals. Here, chatbots can be an alternative to assist pregnant women in managing their mental health^176^ by facilitating cognitive behavioral therapy and psychological support^177–179^ and providing antenatal recommendations.^180^ However, more research is needed to design chatbots that are safe for patients^181^ by leveraging existing design guidelines^182^ (e.g. clarity around capabilities, sustaining conversations, handling dialog failures) and accounting for the socio-technical nuances in the design of human–chatbot interactions.^183^

Moreover, our study shows how healthcare professional's efforts dealing with infrastructural constraints (e.g. space, material, etc.) are often perceived as a delay in the time of the consultation, a lack of empathy (e.g. negative attitudes), or a lack of quality affecting women's acceptance and utilization of antenatal services. Similar to a recent study in Nepal,^142^ our participants utilized private antenatal services more as they were perceived to have better quality in terms of health facilities and attention from healthcare professionals. In addition, the technical constraints (e.g. system failures) resulted in different adverse effects, including additional technology-mediated work, increasing the risk of burnout.^184^ To address these issues, our findings suggest that future digital health tools for antenatal care should support healthcare professionals’ well-being by minimizing their cognitive work (e.g. information overload, task frustration), decision fatigue, and the complexity of their everyday clinical tasks.^185^ For example, the use of natural language processing tools to analyze information from free-text narratives^184^ can help reduce the time spent on paper-based work (e.g. entering information) and enable more time to focus on their patients, which can help to reduce the risk of stress and burnout.^186^ While some healthcare professionals expressed their need to receive help and learn how to manage stress, unfortunately, mental health support for healthcare professionals is limited or not even available in public health institutions in our study. As it could be difficult for healthcare professionals to balance their emotional involvement while managing different shifts, patients, and complications, our study highlights the need to design digital health tools that can enhance the coordination of inter-professional care plans,^185^ the provision of peer support and intercollegiate counseling groups,^187^ and facilitate the provision of training for empathy,^188^ going beyond monitoring and supervision.^189^ Indeed, peer-to-peer strategies can offer a safe space to discuss healthcare professionals’ feelings and emotional concerns and help prevent or reduce burnout. Other technologies, such as fitness apps, meditation, breathing applications, or self-help programs, can also provide healthcare professionals with individual stress management strategies that can help remove the stigma of therapy using digital resources.^190^ However, structural problems such as lack of human, physical, and technical infrastructure must also be addressed to tackle what is causing stress in the first place, especially in the public health subcenters where most of these issues were reported.

### Exploring the use of sociomaterial approaches in the design of digital maternal health

Our study shows the importance of understanding the complex socio-material dynamics of ANCs and how these affect women's care experiences, aligned with the more-than-human perspective of digital health.^191^ Indeed, recent work has highlighted how digital health is often disconnected from the complexities of healthcare settings^192^ and the need for taking a socio-ecological approach^92,94,193^ to understand and design technologies in the context where multifaceted and interconnected elements (e.g. people, physical artifacts, digital systems, spaces, routines, etc.) shape pregnancy care experiences.

In LMICs, there is limited understanding of how the local ecosystem and existing social and material agencies and practices can impact the implementation and adoption of healthcare interventions,^194^ especially around maternal health.^68,121^ All the material (e.g. lack of medications or ultrasound devices), technical (e.g. lack of power, systems failures), spatial (e.g. small spaces and lack of hygiene in healthcare environments), organizational and everyday life constraints (e.g. short time of consultations, everyday life activities) reported in our findings can result in unintended health consequences and influence the design, implementation, and use of digital health during pregnancy complications. There is a need for taking a socio-material approach to understand antenatal care practices and scope opportunities for digital health.^92–94^ Here, we can combine human factors and a social science approach^195^ to further understand multiple stakeholder's needs. This includes an understanding of pregnant women and healthcare professionals’ experiences, challenges and invisible work practices, using human-centered design^196^ and participatory design^171^ approaches to ensure their voices are heard in the design of digital maternal health interventions.^68^ Failing to recognize the socio-technical context and the complexity of healthcare practices and everyday pregnant women lived experiences in technology design can result in technology having low impact^197^ as it would not fit into people's lives and routines^198^ favoring a “one size fits all” solution that would not work in practice.^192^ Although our findings have implications for the design of digital health interventions, they are far from complete, and future work should continue developing our understanding of antenatal care and digital health practices in context,^192^ emphasizing the complex socio-material arrangements in pregnancy care.

### Limitations

Our exploratory study is situated within an interpretive research paradigm and followed a reflexive practice of knowledge generation. As such, we recognise the potential for further theme development, new insights, or understanding beyond our analysis that was dependent on the situated views of the research teams rather than providing a full description of all aspects of the phenomenon under study.^102,104^ In particular, our study has the following limitations. First, even though we used purposeful sampling to recruit participants with complications through hospitals and healthcare subcenters to get a contextual understanding of their challenges accessing antenatal services from three different geographical locations, our study was conducted only in Ecuador. Future studies should investigate the challenges and experiences of pregnant women accessing antenatal services during complications in other geographical regions in Ecuador and LATAM. Second, although all participants read the participant information sheet and provided consent to participate in the study, we noticed that few participants limited their responses due to fear of retaliation by medical or legal authorities in the case of healthcare professionals and by healthcare professionals in the case of pregnant women. At different moments, we reaffirmed participants that their participation was anonymous, which facilitated conducting both interviews and focus groups. Third, our exploratory study included a large and diverse sample for a qualitative study^199^ but did not separate participants by type of complication, age, or socioeconomic status, and it did not involve policy makers. Future studies should explore additional patterns of pregnant women's experiences as part of the same homogeneous group of participants (e.g. same complication or age group) to enrich the data collection of lived experiences. In addition, future work should seek to involve policy makers in investigating the social determinants of pregnancy complications as well as in the design of digital health interventions for maternal health. Lastly, the Ecuadorean technology market has recently experienced sustained growth due to increased access to smartphones (urban from 54% in 2019 to 64.9% in 2024; rural from 28.8% in 2019 to 42% in 2024) and internet usage (urban from 66.7% in 2019 to 85.1% in 2024; rural from 42.9% in 2019 to 59.8% in 2024).^200^ While this will enable the development of digital health services, especially in the context of antenatal care, this will also require collaborative and culturally relevant research and training programs in digital health.^201^ Future studies should also investigate the challenges that non-mobile phone users and other minority groups (e.g. migrants, people with disabilities, etc.) face while accessing antenatal services during complications.

## Conclusion

Digital health tools hold significant promise for improving the limited access to and utilization of ANCs in Latin America. Understanding the socio-material dynamics is essential for the design of effective digital maternal health interventions. This study offers insights into many context-specific socio-technical challenges and constraints that impact the access and use of antenatal services, particularly in cases of pregnancy complications in Ecuador. Our study also presents insights about the potential of digital health tools to support patients and healthcare professionals in managing pregnancy complications, highlighting the importance of taking a socio-material approach to identify barriers and opportunities for the equitable implementation of digital health in antenatal care. Future research should include a broader range of regions in Ecuador and Latin America while actively engaging pregnant women, healthcare professionals, and policy makers in investigating social determinants that influence pregnancy complications that could inform the development of contextually appropriate digital health interventions for antenatal care. Further research with non-mobile phone users and other vulnerable communities, including migrants and individuals with disabilities, in the maternal health is needed.

## Supplemental Material

sj-pdf-1-dhj-10.1177_20552076251343684 - Supplemental material for Socio-technical challenges in accessing antenatal services during pregnancy complications in Ecuador and the opportunities for digital health Supplemental material, sj-pdf-1-dhj-10.1177_20552076251343684 for Socio-technical challenges in accessing antenatal services during pregnancy complications in Ecuador and the opportunities for digital health by Lorena Carlo, Eduardo Cruz, Francisca Antonella Carpio-Arias, Martin Baca, Maria Paula Jaramillo Carlo, Valeria Carpio-Arias, William Waters, Nicola Mackintosh and Nervo Verdezoto in DIGITAL HEALTH

sj-docx-2-dhj-10.1177_20552076251343684 - Supplemental material for Socio-technical challenges in accessing antenatal services during pregnancy complications in Ecuador and the opportunities for digital health Supplemental material, sj-docx-2-dhj-10.1177_20552076251343684 for Socio-technical challenges in accessing antenatal services during pregnancy complications in Ecuador and the opportunities for digital health by Lorena Carlo, Eduardo Cruz, Francisca Antonella Carpio-Arias, Martin Baca, Maria Paula Jaramillo Carlo, Valeria Carpio-Arias, William Waters, Nicola Mackintosh and Nervo Verdezoto in DIGITAL HEALTH

sj-docx-3-dhj-10.1177_20552076251343684 - Supplemental material for Socio-technical challenges in accessing antenatal services during pregnancy complications in Ecuador and the opportunities for digital health Supplemental material, sj-docx-3-dhj-10.1177_20552076251343684 for Socio-technical challenges in accessing antenatal services during pregnancy complications in Ecuador and the opportunities for digital health by Lorena Carlo, Eduardo Cruz, Francisca Antonella Carpio-Arias, Martin Baca, Maria Paula Jaramillo Carlo, Valeria Carpio-Arias, William Waters, Nicola Mackintosh and Nervo Verdezoto in DIGITAL HEALTH

sj-docx-4-dhj-10.1177_20552076251343684 - Supplemental material for Socio-technical challenges in accessing antenatal services during pregnancy complications in Ecuador and the opportunities for digital health Supplemental material, sj-docx-4-dhj-10.1177_20552076251343684 for Socio-technical challenges in accessing antenatal services during pregnancy complications in Ecuador and the opportunities for digital health by Lorena Carlo, Eduardo Cruz, Francisca Antonella Carpio-Arias, Martin Baca, Maria Paula Jaramillo Carlo, Valeria Carpio-Arias, William Waters, Nicola Mackintosh and Nervo Verdezoto in DIGITAL HEALTH

sj-docx-5-dhj-10.1177_20552076251343684 - Supplemental material for Socio-technical challenges in accessing antenatal services during pregnancy complications in Ecuador and the opportunities for digital health Supplemental material, sj-docx-5-dhj-10.1177_20552076251343684 for Socio-technical challenges in accessing antenatal services during pregnancy complications in Ecuador and the opportunities for digital health by Lorena Carlo, Eduardo Cruz, Francisca Antonella Carpio-Arias, Martin Baca, Maria Paula Jaramillo Carlo, Valeria Carpio-Arias, William Waters, Nicola Mackintosh and Nervo Verdezoto in DIGITAL HEALTH
