# Supplementary material for: Socio-technical challenges in accessing antenatal services during pregnancy complications in Ecuador and the opportunities for digital health
Source: Digit Health. 2025 Jun 9;11:20552076251343684. doi: 10.1177/20552076251343684 (PMC12159480; doi:10.1177/20552076251343684)
Supplement: sj-docx-2-dhj-10.1177_20552076251343684 - Supplemental material for Socio-technical challenges in accessing antenatal services during pregnancy complications in Ecuador and the opportunities for digital health [file sj-docx-2-dhj-10.1177_20552076251343684.docx]

Women’s Everyday Experiences and Infrastructural Challenges of Healthcare Systems in Relation to Maternal and Neonatal Health to Scope Opportunities for Digital Health Technologies in Pichincha, Guayas, and Chimborazo, 2019

**Focus Group Discussion Guide**

**Introduction**

Welcome. Thank you for joining this meeting, whose purpose is to have a conversation about your experiences and opinions regarding your pregnancy and the use of prenatal healthcare services. You have been invited because you are pregnant and because some complications have been identified in your pregnancy.

We hope that during this conversation, everyone present can contribute their ideas. We will not take turns speaking. Instead, it is a conversation among friends. All opinions are welcome. There are no right or wrong answers. You may agree or disagree with the ideas that arise, but at all times, we will respect each other's opinions.

It is important that only one person speaks at a time so that we can clearly hear what each person has to say. Additionally, to properly record your ideas, this conversation will be recorded. If many people speak at once, the recording will not be clear.

During the discussion, we will use only your first names. Your identity will not be revealed at any time. Your ideas and opinions are confidential. We appreciate your help in filling out the informed consent form that we are distributing.

During the conversation, we will provide refreshments and something healthy to eat.

**Warm-up: Participants introduce themselves and mention something about their family, starting with the moderator.**

1. First, I would like to ask you about your experiences with your pregnancy.

a. Tell us about this pregnancy. How has it been for you?

b. Has your partner helped you with the pregnancy? How? Have other people helped you?

c. Has your pregnancy been easy or difficult? How do you feel?

1. During your pregnancy, have you used any practices that come from your beliefs or the beliefs of your family or community?
2. Let's talk a bit about the complications you have had during your pregnancy.

a. What complications have been identified or diagnosed? How and when were they diagnosed? What was your reaction to the diagnosis?

b. What do you think about your complications? Do you have any concerns about your baby or yourself? How do you feel emotionally?

c. Have you shared these complications with others (for example, family, friends, community members, etc.)?

d. How has your life changed due to your pregnancy and complications? What do you do in your daily life to manage these complications and their impact on your personal, family, or social life?

1. Now, let’s talk about healthcare services. First, where do you go for the prenatal healthcare services you need? Are they public or private hospitals and/or health centers?

a. What factors do you consider when deciding to seek, access, and choose the type of institution or healthcare provider you go to? Does anyone help you in the search or decision-making process?

1. How has your experience been with prenatal care at the centers or hospitals you visit?

a. Have you had issues with costs or insurance? What has been your experience with appointments or scheduling?

b. How do doctors and nurses treat you? Are you satisfied with the treatment?

c. How many doctors do you interact with at the center or hospital? Do you trust the diagnosis and treatment received, or have there been any issues? Have you seen improvements? Have you changed doctors? Why?

d. Based on your experience, do the environment, physical infrastructure, and medical staff of the healthcare providers you visit influence your behavior, perception, and need for care?

1. Now, I would like to ask about the availability of information and knowledge you have obtained about your pregnancy and complications.

a. Have they explained to you how to manage your pregnancy and the risks of your complications? What have you learned? Who has informed you? Do you understand well what they have told you? Do you have any difficulty following the instructions and treatments given in medical prescriptions?

b. How could the understanding of information and your complications be improved to enhance your health during pregnancy?

c. Have you felt the need or sought information elsewhere about your complications? Where or through what means have you obtained this information? Has it been helpful or not? What problems did you encounter?

d. Would you like to receive information through your mobile phones? Can you think of any technologies that could help you manage your conditions more effectively?

1. Is there any other topic you would like to mention?
