# Supplementary material for: Socio-technical challenges in accessing antenatal services during pregnancy complications in Ecuador and the opportunities for digital health
Source: Digit Health. 2025 Jun 9;11:20552076251343684. doi: 10.1177/20552076251343684 (PMC12159480; doi:10.1177/20552076251343684)
Supplement: sj-docx-3-dhj-10.1177_20552076251343684 - Supplemental material for Socio-technical challenges in accessing antenatal services during pregnancy complications in Ecuador and the opportunities for digital health [file sj-docx-3-dhj-10.1177_20552076251343684.docx]

**Health Services for Pregnant Women with Complications**

**Guide for Individual Interview Questions**

Good morning/afternoon. Thank you for your collaboration with our study on prenatal health services received by women with pregnancy complications. This study is part of a collaboration between several universities: Escuela Superior Politécnica del Litoral, Escuela Superior Politécnica de Chimborazo, and Universidad San Francisco de Quito in Ecuador, and the University of Leicester in the United Kingdom.

This conversation will last approximately half an hour and will focus on your opinions and perceptions as a healthcare professional. Everything you share with us is confidential. Your name or identity will not be included in the study. You may decide not to participate and can stop at any time. We appreciate your help in completing the informed consent form we are distributing.

We will now proceed with the first question. Do you agree?

1. First, can you describe the most important activities or responsibilities you have regarding the care of pregnant women with complications?

a. How would you describe your work, and what challenges do you experience in the daily healthcare of pregnant women with complications?

b. What do you think about your workload? How long is your shift? Do you think you dedicate enough time to each patient?

c. Do you receive continuous training at your institution? Do you generally feel that your work is valued by your patients and the administrators of the healthcare institution you work for?

d. Do you think you need or have received any kind of emotional support?

1. How would you describe your patients, specifically pregnant women with complications?

a. What barriers exist in the care of pregnant women with complications? How could citizen involvement and participation in this important issue be improved?

b. Have you encountered pregnant patients whose socio-cultural customs/beliefs conflict with standard health services? What are those beliefs and issues?

1. How would you describe the physical and human infrastructure of your hospital or health center?

a. In your experience, are there differences in infrastructure among different hospitals and health centers?

b. Are there differences in terms of equipment, supplies, physical space, and aesthetics (sound, lighting, odors)? How does this influence/affect your work?

b. Are there any local, institutional, or national regulations or rules that you feel positively or negatively influence or affect your work?

c. How would you describe the work environment with administrative and healthcare personnel at your institution? How would you describe the treatment and care patients receive daily?

d. What is the medical record system in the hospital or health center? Do you have access to the medical record systems of other institutions at the local or national level? How and when do you use them in relation to the care of pregnant women with complications? Is it useful to you or not?

e. What other technologies are used in your institution that you have found useful or not useful?

f. During the process of caring for pregnant women with complications: Have you experienced system failures? Have you had any problems with equipment failures or a lack of resources? How were these problems resolved?

g. Do you have a smartphone? Do you use your mobile phone or any technological application during your professional practice?

1. How would you characterize the communication between healthcare professionals collaborating on a specific case and between healthcare professionals and patients with pregnancy complications?

a. How many healthcare professionals do you collaborate with daily to care for pregnant women with complications? Who are these professionals?

b. Do you use tools (physical or digital) or strategies that support the coordination of healthcare for pregnant women with complications in your institution?

1. What do you think about the incorporation of digital information and systems into healthcare services?

a. What types of technologies do you think could be used to improve maternal health service delivery in your institution?

b. What types of technologies do you think could be incorporated to improve the self-care of health for pregnant women with complications?

c. In your experience, do you have any concerns regarding the use of technology by pregnant women with complications in relation to health self-care?

1. Is there anything else you would like to mention?
