# Supplementary material for: Socio-technical challenges in accessing antenatal services during pregnancy complications in Ecuador and the opportunities for digital health
Source: Digit Health. 2025 Jun 9;11:20552076251343684. doi: 10.1177/20552076251343684 (PMC12159480; doi:10.1177/20552076251343684)
Supplement: sj-docx-4-dhj-10.1177_20552076251343684 - Supplemental material for Socio-technical challenges in accessing antenatal services during pregnancy complications in Ecuador and the opportunities for digital health [file sj-docx-4-dhj-10.1177_20552076251343684.docx]

Women’s Everyday Experiences and Infrastructural Challenges of Healthcare Systems in Relation to Maternal and Neonatal Health to Scope Opportunities for Digital Health Technologies in Pichincha, Guayas and Chimborazo, 2019

**Guía de preguntas para grupos focales**

**Introducción**

Bienvenidas. Gracias por venir a esta reunión, cuyo propósito es tener una conversación sobre sus experiencias y opiniones en cuanto a su embarazo y el uso de servicios de salud prenatal. Ustedes han sido invitadas porque están embarazadas y porque se ha identificado alguna complicación en su embarazo.

Esperamos que durante esta conversación todas las personas presentes puedan aportar con sus ideas. No vamos a hablar por turnos. Más bien, es una conversación entre amigas. Todas las opiniones son bienvenidas. No existen opiniones o respuestas buenas o malas. Se puede estar en acuerdo o en desacuerdo con las ideas que surjan, pero en todo momento vamos a respetar las opiniones de los demás.

Es importante que sólo una persona hable a la vez, de esta manera escucharemos bien lo que cada persona va a decir. Además, para llevar un registro adecuado de sus ideas, esta conversación será grabada. Si muchas personas hablan al mismo tiempo no se podrá escuchar bien la grabación.

Durante la discusión vamos a usar solamente sus primeros nombres. No vamos a revelar su identidad en ningún momento. Sus ideas y opiniones son confidenciales. Agradecemos que nos ayuden llenando el formulario de consentimiento informado que estamos distribuyendo.

Durante la conversación les estaremos ofreciendo refrescos y algo saludable para comer.

**Calentamiento: Las participantes se presentan y menciona algo sobre su familia, comenzando con la moderadora.**

1. Primero quiero preguntarles sobre sus experiencias el su embarazo.

a. Cuéntenos sobre este embarazo. ¿Cómo les ha ido?

b. ¿Su pareja les ha ayudado con el embarazo? ¿Cómo? ¿Otras personas les han ayudado?

c. ¿Su embarazo ha sido fácil o difícil? ¿Cómo se sienten?

2. ¿Durante su embarazo ustedes han usado alguna práctica que viene de sus creencias o las creencias de sus familiares o de sus comunidades?

3. Conversemos un poco sobre las complicaciones que han tenido en sus embarazos.

a. ¿Qué complicaciones les han identificado o diagnosticado? ¿Cómo y cuándo se las diagnosticaron? ¿Cuál fue su reacción frente al diagnóstico?

b. ¿Qué piensan de sus complicaciones? ¿Tienen alguna preocupación sobre su bebé o sobre ustedes? ¿Cómo se sienten emocionalmente?

c. ¿Han compartido estas complicaciones con otros (por ejemplo familia, amigos, miembros de comunidad, etc.)?

d. ¿Cómo han cambiado sus vidas debido a su embarazo y complicaciones? ¿Qué hacen en la vida cotidiana para manejar estas complicaciones y el impacto que tienen en su vida personal, familiar o social?

4. Ahora vamos a conversar sobre los servicios de salud. Primero, ¿a dónde van para los servicios de salud prenatal que necesitan? ¿Son hospitales y/o centros de salud públicos o privados?

a. ¿Qué factores toman en cuenta al tomar la decisión de buscar, acceder y escoger el tipo de institución o proveedor de salud al que van? ¿Alguien les ayuda en la búsqueda o en la toma de decisiones?

5. ¿Cómo les ha ido en el cuidado de salud prenatal en los centros o hospitales a dónde van?

a. ¿Han tenido problemas con los costos o los seguros? ¿Cuál ha sido su experiencia con citas o turnos?

b. ¿Cómo les tratan los médicos y enfermeras? ¿Están satisfechas con el trato?

c. ¿Con cuantos médicos interactúan en el centro u hospital? ¿Confían en el diagnóstico y tratamiento recibido o ha habido algún problema? ¿Han visto mejorías? ¿Han cambiado de médicos? ¿Por qué?

d. En su experiencia, el ambiente, la estructura física y el personal médico de los proveedores de salud al que asisten tienen alguna influencia en su comportamiento, percepción y necesidad de atención?

6. Ahora, quiero preguntarles sobre la disponibilidad de información y conocimiento que han obtenido sobre sus embarazos y complicaciones.

a. ¿Les han explicado cómo manejar sus embarazos y los riesgos de sus complicaciones? ¿Qué han aprendido? ¿Quién les ha informado? ¿Entienden bien lo que les han informado? ¿Tiene alguna dificultad en seguir las indicaciones y tratamientos dados en las prescripciones médicas?

b. ¿Cómo se podría mejorar la comprensión de la información y de sus complicaciones para mejorar su estado de salud durante el embarazo?

c. Han sentido la necesidad o han buscado información en otros lugares acerca de sus complicaciones? ¿En qué lugares o medios han conseguido dicha información? ¿Les ha servido de ayuda o no? ¿Qué problemas encontraron?

c. ¿Les gustaría recibir información por medio de sus teléfonos celulares? ¿Pueden pensar en tecnologías que podrían ayudarles a manejar sus condiciones de una mejor manera?

7. ¿Hay algún otro tema que quieren mencionar?
