# Supplementary material for: Socio-technical challenges in accessing antenatal services during pregnancy complications in Ecuador and the opportunities for digital health
Source: Digit Health. 2025 Jun 9;11:20552076251343684. doi: 10.1177/20552076251343684 (PMC12159480; doi:10.1177/20552076251343684)
Supplement: sj-docx-5-dhj-10.1177_20552076251343684 - Supplemental material for Socio-technical challenges in accessing antenatal services during pregnancy complications in Ecuador and the opportunities for digital health [file sj-docx-5-dhj-10.1177_20552076251343684.docx]

**Servicios de salud para mujeres embarazadas con complicaciones**

**Guía de preguntas para entrevistas individuales**

Buenos días/buenas tardes. Gracias por su colaboración con nuestro estudio sobre los servicios de salud prenatal que reciben mujeres con complicaciones en el embarazo. El estudio es parte de una colaboración entre varias universidades: Escuela Superior Politécnica del Litoral, Escuela Superior Politécnica de Chimborazo y la Universidad San Francisco de Quito, en el Ecuador, y la Universidad de Leicester en el Reino Unido.

La conversación tendrá aproximadamente media hora y trata sobre sus opiniones y percepciones como profesional en la salud. Todo lo que usted comparte con nosotros es confidencial. No se va a incluir su nombre o identidad en el estudio. Usted puede decidir no participar y puede dejar de participar en cualquier momento. Agradecemos que nos ayuden llenando el formulario de consentimiento informado que estamos distribuyendo.

Vamos a proceder con la primera pregunta. ¿Está usted de acuerdo?

1. Primero, ¿puede describir las actividades o responsabilidades más importantes que tiene usted en cuanto al cuidado de mujeres embarazadas con complicaciones?

a. ¿Cómo describiría su trabajo y cuáles son las dificultades que experimenta en el cuidado diario de la salud de mujeres embarazadas con complicaciones?

b. ¿Qué opina usted de su carga de trabajo? ¿De cuantas horas es su turno? ¿Usted piensa que le dedica tiempo suficiente a cada paciente?

c. ¿Recibe entrenamiento continuo en su institución? ¿En general se siente que su trabajo es valorado por sus pacientes y los administradores de la institución de salud para la que trabaja?

d. ¿Usted piensa que necesita o han recibido algún tipo de soporte emocional?

2. ¿Cómo describe usted a sus pacientes, específicamente mujeres embarazadas con complicaciones?

a. ¿Cuáles son las barreras existentes en el cuidado de las mujeres embarazadas con complicaciones? ¿Cómo se podría mejorar el involucramiento y participación de la ciudadanía en este tema tan importante?

b. ¿Ha encontrado a pacientes embarazadas cuyas costumbres/creencias socio-culturales estén en conflicto con los servicios estándares de salud? ¿Cuáles son esas creencias y problemas?

3. ¿Cómo podría describir la infraestructura física y humana que tiene su hospital o centro de salud?

a. En su experiencia, ¿hay diferencias en infraestructura en diferentes hospitales y centros de salud?

b: ¿Hay diferencias en cuanto a equipos, insumos, espacio físico, estética del lugar (sonido, iluminación, olores)? ¿Cómo influencia/afecta esto a su trabajo?

b. ¿Hay alguna normativa local, institucional o nacional o reglas que usted siente que influencia o afecta positivamente o negativamente su trabajo?

c. ¿Cómo describe usted el ambiente de trabajo con el personal administrativo y de salud de su institución? ¿Cómo describe su trato y atención a los pacientes que recibe diariamente?

d. ¿Cuál es el sistema de registros médicos en el hospital o centro de salud? ¿Tiene acceso a los sistemas de registros médicos de otras instituciones a nivel local o nacional? ¿Cómo y cuándo los usa en relación con el cuidado de mujeres embarazadas con complicaciones? ¿Le es útil o no?

e. ¿Qué otras tecnologías usan en su institución, que le hayan parecido útiles o no útiles?

f. Durante el proceso de cuidado de mujeres embarazadas con complicaciones: ¿Ha experimentado la caída del sistema informático? ¿Ha tenido algún problema con la falla de algún equipo o le ha faltado algún recurso? ¿Cómo resolvieron estos problemas?

g. ¿Tiene usted Smartphone? Utiliza su teléfono móvil o alguna aplicación tecnológica durante su ejercicio profesional?

4. ¿Cómo caracterizaría la comunicación entre profesionales de salud que colaboran en un caso específico y entre profesionales de salud y pacientes con complicaciones de embarazo?

a. ¿Con cuántos profesionales de salud colabora diariamente para atender a mujeres embarazadas con complicaciones? ¿Quiénes son estos profesionales?

b. ¿Usan herramientas (física o digital) o estrategias que apoyen la coordinación del cuidado de la salud de mujeres embarazadas con complicaciones en su institución?

5. ¿Qué opina usted sobre la incorporación de información y sistemas digitales en los servicios de salud?

a. ¿Qué tipo de tecnologías piensa que se pueden usar para mejorar la prestación de servicios de salud materna en su institución?

b. ¿Qué tipo de tecnologías cree que podrían ser incorporadas para mejorar el autocuidado de la salud de mujeres embarazadas con complicaciones?

c. En su experiencia, ¿tiene alguna preocupación con respecto al uso de tecnologías por parte de las mujeres embarazadas con complicaciones en relación al autocuidado de salud?

6.¿ Hay algo más que quiere mencionar?
